# Prevalence and risk factors associated with cryptosporidiosis among children within the ages 0–5 years attending the Limbe regional hospital, southwest region, Cameroon

**DOI:** 10.1186/s12889-019-7484-8

**Published:** 2019-08-20

**Authors:** Atsimbom Neville Tombang, Ngwa Fabrice Ambe, Tanyi Pride Bobga, Claude Ngwayu Nkfusai, Ngandeu Mongoue Collins, Sangwe Bertrand Ngwa, Ngwene Hycentha Diengou, Samuel Nambile Cumber

**Affiliations:** 1 Department of Medical Laboratory Sciences, Faculty Health Sciences, University of Buea, Buea, Cameroon; 2 Department of Microbiology and Parasitology, Faculty of Science, University of Buea, Buea, Cameroon; 3 Cameroon Baptist Convention Health Service (CBCHS), Yaoundé, Cameroon; 4 Center for Medical Research, Institute of Medical Research and Medicinal Plant Studies, Ministry of Scientific Research and Innovation, Yaounde, Cameroon; 5 Faculty of Health Sciences, University of the Free State, Bloemfontein, South Africa; 6 Section for Epidemiology and Social Medicine, Department of Public Health, Institute of Medicine, The Sahlgrenska Academy at University of Gothenburg, Gothenburg, Sweden; 7 School of Health Systems and Public Health, Faculty of Health Sciences, University of Pretoria Private Bag X323, Gezina, Pretoria, 0001 Pretoria, South Africa

**Keywords:** Cryptosporidiosis, Prevalence, Risk factors, Children 0–5 years, Limbe

## Abstract

**Background:**

Cryptosporidiosis is a pathological condition caused by infection with coccidian protozoan parasites *Cryptosporidium*. *Cryptosporidium* is one of the most common causes of childhood diarrhea in developing countries. So far, no data has been published on its prevalence among children with diarrhea in Cameroon. This study was therefore, designed to assess the prevalence and risk factors associated with Cryptosporidiosis among children within the ages 0–5 years suffering from diarrhea and being attended to at the Limbe Regional Hospital.

**Methods:**

The study was a hospital based analytical cross-sectional study involving children within the ages 0–5 years (*n* = 112) hospitalized or consulted in the pediatric departments of the hospital between April 2018 and May 2018. Stool specimens were processed using the modified acid-fast staining method, and microscopically examined for *Cryptosporidium* infection.

**Results:**

A total of 112 participants were recruited out of which 67 presented with diarrhea. A high prevalence 9/67 (13.40%) of *Cryptosporidium* was noticed in children with diarrhea than children without diarrhea 1/45 (2.2%). There was a significant relationship (***p*** **= 0.041**) between prevalence of *Cryptosporidium* and the presence of diarrhea in children within the ages 0–5 years in the Limbe Regional Hospital. It was realized that children from parents with primary level of education, children whose parents did not respect exclusive breastfeeding and those whose parents were giving them pipe borne water for drinking recorded a higher prevalence.

**Conclusions:**

This study revealed an overall prevalence of 8.9% for *Cryptosporidium* among children of ages 0–5 years that attended the Limbe Regional Hospital. The prevalence among children that presented with diarrhea was 13.4%. The study clearly demonstrated that *Cryptosporidium* is an important protozoal etiologic agent for children with diarrhea in Limbe.

**Electronic supplementary material:**

The online version of this article (10.1186/s12889-019-7484-8) contains supplementary material, which is available to authorized users.

## Background

Human cryptosporidiosis is caused by infection with apicomplexan protozoans of the genus *Cryptosporidium*. *Cryptosporidium* causes diarrheal disease in humans worldwide [1]. In the 1980s, *Cryptosporidium* was recognized as an important cause of persistent diarrhea in immunocompromised patients (AIDS) [2, 3]. The parasite was gradually linked to malnutrition and death caused by diarrhea in children in developing countries [4]. *Cryptosporidium* species is currently a major cause of waterborne outbreaks worldwide, reported in 239 waterborne outbreaks between 2011 and 2016 [5]. Despite the seemingly ubiquitous nature of cryptosporidiosis, sufficient attention has not been paid to it, prompting the WHO in 2004 to list it among globally “neglected diseases” which have a common link with poverty in most developing countries [6]. Human illness was formerly thought to be caused by a single species, but molecular studies have demonstrated that it is caused by at least 20 different species, among them the *C. hominis* and *C. parvum* are the most reported species [1].

The parasitic protozoa *Cryptosporidium spp.* attracts attention with large epidemics in industrialized countries while being undiagnosed and neglected in many developing countries [7, 8]. *Cryptosporidiosis* account for about 30–50% of deaths in young individuals(infant and children) worldwide and found to be the second leading cause of diarrhea and deaths in children after rotavirus [3].Sub Saharan Africa accounts for half of all global childhood deaths from diarrheal diseases [9] and it has been estimated that over 2.9 million of *Cryptosporidium* infections occur every year in children aged less than 24 months in Sub Saharan Africa [6]. *Cryptosporidium* is known as an opportunist disease-causing, causing diarrhea and intestinal disorders in the immune deficit and immune competent individuals but it is self limiting in immune competent host [10]. Transmission occurs via the fecal-oral route from human and animal reservoirs [11, 12]. However, it has been shown to induce weight loss, growth stunting, sustained impact on child development and increased case fatality [4], and according to WHO, diarrhea accounts for 10.5% of the nearly 8 million yearly deaths of children under 5 years of age [13].

The symptoms of acute cryptosporidiosis include severe watery diarrhea, eventually leading to dehydration, mal-absorption and malnutrition [14, 15]. Earlier prevalence of cryptosporidiosis varied from 1% in high-income countries to 5–10% in low and middle-income countries [1]. Asian countries are among low income countries with high burden of *Cryptosporidium* amongst young people according to Khan in Pakistan (2019) (29.88%) [3] and Aghamolaie in Iran (2016) (1.2%) [14]. According to Nsagha in Fako (2016), Cameroon is among the Sub-Saharan African countries with a very high burden of *Cryptosporidium* amongst HIV infected people, (44.0%) [16]; Bessong in Bamenda (2015, 7.0%) [17]; Vouking in Yaoundé (2014, 7.2%) [18]; Sarfati in West Region (9.7%) [19] revealing that *cryptosporidium* still is a public health challenge among HIV/AIDS patients in Cameroon. An estimated 80% of Acquired Immune Deficiency Syndrome(AIDS) patients die of AIDS-related opportunistic infections rather than from the HIV itself [20, 21]. However, this pathogen is not often sought as etiology during diarrhea in children in Cameroon with limited information on the prevalence of cryptosporidiosis in children. It is based on these research works carried out in Cameroon with its delimitation to HIV infected individuals that we sort to investigate the **“**Prevalence and risk factors associated with Cryptosporidiosis among children within the ages 0-5 years attending the Limbe Regional Hospital, Southwest Region, Cameroon”.

## Methods

### Study area and setting

The study was carried out at the pediatric and laboratory units of the Limbe Regional Hospital located at mile one in Limbe. The pediatric ward in this hospital is the most populated ward in Fako Division in the Southwest Region of Cameroon. This Hospital is serving clients from within and without the Sub-Division. Limbe is the Divisional Head Quarters and one of the major urban centers in Fako Division. Limbe is the head quarter of the Cameroon Development Corporation (CDC), one of the largest employers in Cameroon after the government and also a major touristic site with features such as the beach, botanical garden and the zoo. Almost all ethnic groups in Cameroon are represented in this town attracted by the business friendly environment, education, leisure and job opportunities.

### Study design

This study was a hospital based cross-sectional study conducted at the Limbe Regional Hospital Laboratory between the 10th of April and 30th of June, 2018. Participants hospitalized or consulted in the pediatric department, Infant Welfare Clinic (IWC) or the Outpatient Department were enrolled. Consecutive sampling technique was used, in which case participants who met the inclusion criteria and consented to our study were directly selected as they came.

### Sample size calculation

Sample size was calculated from the Lorenz formula: $\frac{\mathrm{n}={\mathrm{Z}}^2\mathrm{P}\left(1\hbox{-} \mathrm{P}\right)}{{\mathrm{d}}^2}$

Where n is the sample size.

Z is the confidence interval (95%) Z = 1.95.

P is the pre-estimated prevalence of 4.8% obtained from a study carried out by Anejeo et al., in 2016 in Jos, Nigeria [22].

d is the margin error (5%).

n = (1.96)2 × 0.058(1–0.048)/ (0.05)2 = 70.218.

This means, a minimum of 71 participants were to take part in this study.

### Study population

The target population of this research were Children within the ages 0–5 years attending the Limbe Regional Hospital.

### Inclusion criteria

The study was delimited to children within the ages 0–5 years that presented with diarrhea (cases) and without diarrhea (control) irrespective of their social status and Religion whose parents/guardians signed the assent forms for their children after clear explanation of the study objectives both in English, French or Pidgin English.

### Exclusion criteria

The study excluded children above 5 years and some of those children 0–5 years whose parents/guardians did not give their consent by reading and signing the assent forms. Also, children whose guardians could not ascertain their ages were excluded from the study.

### Ethical considerations

An authorization N° 2018/0004/UB/HOD/MLS/FHS, was obtained from the Head of Department of Medical Laboratory Science. This was used to obtained Administrative authorization from the Regional Delegation of Public Health and the Limbe Regional Hospital representing the Ministry of Public Health in the Southwest Region. Also, an ethical clearance for this research was obtained from the Institutional Review Board (IRB) of the Faculty of Health Sciences (FHS), University of Buea. Participation and reporting contained herein was based on assent forms signed by parents/guardians of the participants (children 0–5 years) prior to administration of questionnaire. The respondents were adequately informed using the participant’s information sheet about their rights and all the relevant aspects of the study, including its aim and interview procedure. No minor was older than 5 years.

### Quantitative data collection

#### Administering questionnaire

Questionnaires were used to collect demographic information that included; name, age, sex, clinical symptoms including diarrhea and its duration, patient’s residence, type of toilet facility, sources of drinkable water at home and school etc. Diarrhea was defined as the passage of watery or loose stools three or more times within a 24 h period. Acute diarrhea was defined as diarrhea lasting less than 14 days while persistent diarrhea was diarrhea that lingered for more than 14 days. This questionnaire was adapted from the study by Anejo-Okopi in Nigeria [22]. (Additional file 1).

#### Stool sample collection

Parents/guardians of participants (children) with gastrointestinal symptoms (diarrhea with/or vomiting and without diarrhea) were given labeled stool containers to provide one stool sample on the day of enrollment. They were instructed on how to collect desired quantity of stool in the containers and transport them to the principal investigator or researcher as soon as possible.

#### Stool sample handling and storage

Non powdered gloves were used to handle the stool containers. The samples were verified to know if the desired quantity was collected and also to record the macroscopy of the sample. The codes assigned to the containers were also verified to see if they were still visible and corresponded to that on the questionnaires. The Fresh stool samples were preserved at − 20 °C in the fridge in the laboratory for analyses in badges at the end of each working day.

#### Microscopic analysis staining method using modified Ziehl Neelsen (mZN) slide preparation

The stool samples were removed in badges from the fridge and allowed to attend room temperature before moderately thick fecal smears were made on standard glass microscope slides with the aid of a wooden applicator stick to give both thick and thin areas. The smears were allowed to air dry on drying racks.

##### Method

The slides were placed in multi slide carriers for fixation in methanol for 3 min followed by staining with strong carbol fuchsin for about 15 min. The slide was then thoroughly rinsed in tap water. Minimal decolourisation by agitation in a trough of 1% hydrochloric acid-alcohol (70%) for 15 sec, followed by rinsing in tap water. Counterstain for 30 sec in 1% methylene blue, rinse well, and air dry. The stained slides were examined using 40X and 100X oil immersion magnification. The presence or absence of *oocysts* was recorded.

##### Results

Oocysts are characteristically round or slightly ovoid with a consistent modal size, usually of about 4-6ųm. They are acid-fast but oocyst staining within a smear and between specimens, is very variable, and oocysts vary from unstained to partial red staining and complete staining. The “erythrocyte” stained forms are common, and fully sporulated forms can be found in which red staining crescentic bodies, the sporozoites, can be seen within an unstained oocyst wall. The amount of oocysts was estimated as 1+ (1-10oocysts/smear);2 + (11-50oocysts/smear); and 3 + (>50oocysts/smear).

#### Data management and analysis

All the data collected in the field were keyed in Excel sheet in a password protected computer and only authorized individuals could get access to the data. To identify inconsistencies in the data, consistency checks were done e.g. whether dates of children were greater than 5 years. Duplicate checks were performed and if found, these were removed. The data was exported to Statistical Package for Social Sciences (SPSS) version 21 for data cleaning by running frequencies for the different variables. SPSS version 25, excel and Epi-info version 7.0 software packages were used for statistical analyses. A statistical significant level of 5% was used with a 95% confidence interval. For socio-demographic description of the participants, proportions and frequencies were generated. Continuous variables were summarized using the mean. The prevalence of *Cryptosporidium* was calculated as a proportion of children who tested positive for *Cryptosporidium.* A chi square (*X2*) test of proportion was perform to check for association with *Cryptosporidium* demographic factors. The multivariate logistic regression analysis was used to investigate the characteristics associated with *Cryptosporidium* in children lees than 5 years. All clinical characteristics were analyzed in the univariate logistic regression and all the factors that were statistically significant were taken to the multivariate analyses. Odd ratios and *p*-values were used to determine whether each of the exposure factors has an effect on the prevalence of *Cryptosporidium.*

## Results

### Socio-demographic characteristics of participants

A total of 112 parents and their children within the ages 2–60 months old took part in this study. The mean age of the children was 23.1 (SD = 17.4) months and the median age was 14.5 months. 78 (69.6%) of the children were of the age limits 00–30 months old. Educationally wise, 49 (43.8%), 43 (38.4%) and 20 (17.9%) of their parents went through primary, secondary and university education respectively. More than half 66 (58.9%) of the children were female. Also, as concerns the occupation of parents; 18 (16.1%), 10 (8.9%), 50 (44.6%), 18(16.1%) and 16 (14.3%) were farmers, teachers, business people, house wives and others respectively. Most households had 3–5 (73.4%) persons living in the house. As per the monthly income, 60 (53.6%) of the parents earned 50,000F – 100,000F. 66(58.9%) of the participants lived in houses made of blocks while 46 (41.1%) lived in houses made of planks or wood. A majority 76 (67.9%) of the parents had live in their residences for < 9 years. A total of 67 (59.8%), 43 (38.4%), 2 (1.8%) of the participants rent, live in private homes and camps respectively **(**Table **1****).**

**Table 1:** Summary of the socio-demographic characteristics of the participants

CHARACTERISTIC	FREQUENCY (*n* = 112)	PERCENTAGE (%)
Age of child (months)	Mean ± SD (23.1 ± 17.4)	Range (2–60)
0–30	78	69.6
31–60	34	30.4
Gender		
Male	46	41.1
Female	66	58.9
Level of education of parents		
Primary	49	43.8
Secondary	43	38.4
University	20	17.9
Occupation of parents		
Farmer	18	16.1
Teacher	10	8.9
Business	50	44.6
House wife	18	16.1
Others	16	14.3
Monthly income of parents		
20.000F – 50.000F	43	38.4
51.000F – 100.000F	60	53.6
101.000F – 150.000F	8	7.1
> 150.000F	1	0.9
Housing type		
Block house	66	58.9
Plank house	46	41.1

*SD* = Standard Deviation

### The evolution of diarrhea, frequency and stool consistency from children within the ages 0–5 years in Limbe (*n* = 112)

A total of 67 participants from the 112 presented with diarrhea (watery/mucoid stool) that had lasted ≤14 days (acute) passing stool ≥3 times a day while non-presented with diarrhea >14 days. The remaining 45 participants presented with formed/semi-formed stool **(**Table **2****).**

**Table 2:** Characteristics of stool samples collected from children 0–5 years in Limbe

Variable	Total No examined (%)
Evolution of diarrhea	
Acute	67 (100)
Chronic	0 (00)
Frequency	
$$ {\displaystyle \begin{array}{c}\left|\ge \right.3\\ {}<3\end{array}} $$	$\frac{67(59.8)}{45(40.2)}$
Stool consistency	
Watery/mucoid	67 (59.8)
Formed/semi-formed	45 (40.2)

### Prevalence of Cryptosporidium within age groups and the presence/absence of diarrhea

Table 3 shows the age group 00–30 months that recorded a 6/78 (7.7%) prevalence with respect to a 4/34(11.8%) for the age group 31-60 months.The participants that presented with diarrhea recorded a 9/67(13.4%) prevalence while those that did not present with diarrhea recorded a 1/45 (2.2%) prevalence **(**Table 3**).**

**Table 3:** Prevalence of Cryptosporidium within age group and present of diarrhea

Variable	Frequency	Number Positive	Number of microscopy positive samples for Cryptosporidium spp (%)	Number of microscopy positive samples for Cryptosporidium spp within (%)
Age (months)				
00–30	78	6	5.36	7.7
31–60	34	4	3.57	11.8
Diarrhea				
Yes	67	9	8.04	13.4
No	45	1	0.89	2.2

### Prevalence of Cryptosporidium among children within the ages 0–5 years in Limbe that presented with diarrhea

**Fig. 1:**
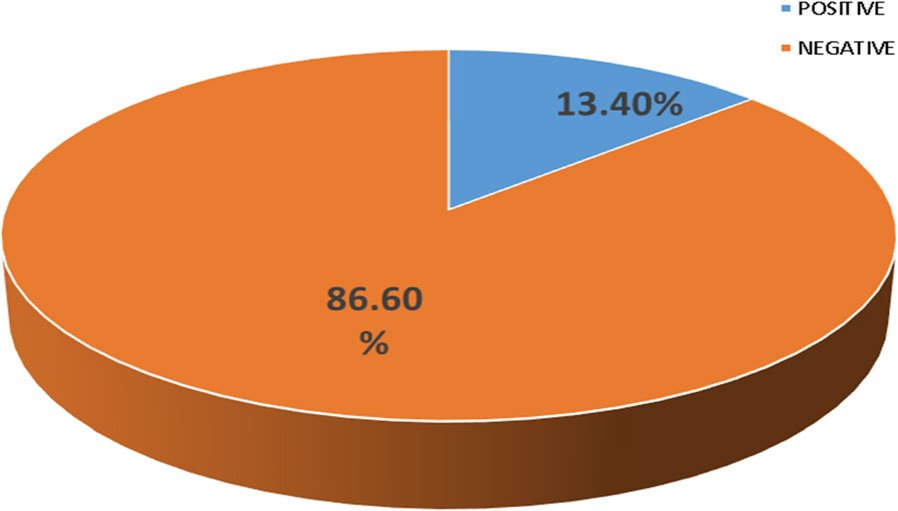
valence of Cryptosporidium among children 0–5 Years in Limbe that Presented with Diarrhea

Figure 1 reveals the prevalence of 9/67 (13.40%) for *Cryptosporidium* among children of ages 0–5 years that presented with diarrhea in the Limbe Regional Hospital. High prevalence (9/67, 13.40%) of *Cryptosporidium* was noticed in children with diarrhea than children without diarrhea (1/45,2.2%). There was a significant association (***p*** **= 0.041**) between prevalence of *Cryptosporidium* and the presence of diarrhea in children of ages 0–5 years in the Limbe Regional Hospital. **(**Fig. 1**).**

### Risk factors associated with cryptosporidiosis in children of ages 00–60 months old

Table 4 summarizes the basic socio-demographic factors associated with cryptosporidiosis in children within the ages 0–5 years which includes; age, gender, level of education of parents, occupation of parents, duration in the locality, housing type, child schooling and the type of dwelling place. Based on host factors, high prevalence 6(5.36%) of cryptosporidiosis was noticed in the age group 00–30 months old though was not statistically significant (*p* = 0.879). With gender, females had high prevalence 6(5.36%) of cryptosporidiosis than males 4 (3.57%), but no significant difference (*p* = 0.942). Also, high prevalence 9 (13.40%) of cryptosporidiosis was noticed in children who presented with diarrhea days before their stool samples were collected compared to children that never had diarrhea 1 (2.2%). Children that were not schooling had high prevalence 7 (6.25%) than those who were schooling 3 (2.68%), though no significant difference (*p* = 0.969).

**Table 4:** Prevalence of cryptosporidium in children 0–5 years old in Limbe based on socio-demographic, host and family factors

Variable	No. sample for each category	No. (100%) positive of cryptosporidium based on categories	Prevalence (8.93%) for each categories	Od ds ratio	95% CI	*p*- value
Age of child						
00–30 months	78 (69.6)	6 (60)	6 (5.36)	1		0.879
30–60 months	34 (30.4)	4 (40)	4 (3.57)	2.52	0.68–9.35	
Gender						
Male	46 (41.1)	4 (40)	(4) 3.57	1		0.942
Female	66 (58.9)	6 (60)	(6) 5.36	0.67	0.18–2.47	
Level of education of parents						
Primary	49 (43.8)	8 (80)	8 (7.14)	1		
Secondary	43 (38.4)	2 (20)	2 (1.79)	0.25	0.05–0.95	0.044
University	20 (17.9)	00	00	0.00	0.0- > 1.0E12	
Occupation of parent						
Teacher	10 (8.9)	00	00			0.150
Business	50 (44.6)	2 (20)	2 (1.79)			
House wife	18 (16.1)	2 (20)	2 (1.79)			
Farmer	18 (16.1)	4 (40)	4 (3.57)			
Others	16 (14.3)	2 (20)	2 (1.79)			
Duration in the locality						
≤ 9 years	76 (67.9)	7 (70)	7 (6.25)	1		0.879
> 10	36 (32.1)	3 (30)	3 (2.68)	0.89	0.22–3.69	
Housing type						
Block house	66 (58.9)	5 (50)	5 (4.46)	1		0.458
Plank house	46 (41.1)	5 (50)	5 (4.46)	1.48	0.41–5.47	
Types of dwelling						
Private house	43	5 (50)	5 (4.46)	1		0.684
Rented	67	5 (50)	5 (4.46)	0.61	0.17–2.26	
Camp house	2	00	00	2.01	0.67–5.23	
Child schooling						
Yes	33 (29.5)	3 (30)	3 (2.68)	1		0.969
No	79 (70.5)	7 (20)	7 (6.25)	1.68	0.44–6.39	

Based on socio-demographic factors, there was a significant difference between the prevalence of cryptosporidiosis and parental level of education. High prevalence 8(7.14%) was seen in children whose parents had primary education than in children whose parents had secondary and university education (2 (1.79%) and 0 (00%) respectively). Other socio-demographic and family factors such as parental occupation, housing type and types of dwelling show no significant difference with prevalence of cryptosporidiosis **(**Table 4**).**

Hygiene, diet and animal risk factors were summarized in Table 5. A significant difference was noticed between the prevalence of *Cryptosporidium* in children and their source of drinkable water at home. High prevalence 8 (7.14%) was seen in children who drank municipal water from tap **(*p*** **= 0.018)** than in children who drank commercial bottled water and municipal water processed at home by filter 1 (0.89%). Also, a significant difference was noticed in prevalence of *Cryptosporidium* in children and mode of nutrition within the first 6 months. Children that never had exclusive breast feeding within the first 6 months of life showed high prevalence 8 (7.14%) of Cryptosporidiosis **(*p*** **= 0.022)** than children who had exclusive breast feeding 2(1.79%). There was no significant association with type of toilet, consumption of beverages, and animal rearing **(**Table 5**).**

**Table 5:** Prevalence of Cryptosporidium in children 0–5 years old in Limbe classified based on hygiene, diet habit and animal factor

Variable	No. sample for each category	No. (100%) positive of cryptosporidium based on categories	Number of microscopy positive samples for Cryptosporidium spp (%)	Odds ratio	95% CI	*P* - value
Type of toilet						
Flushing toilet	8	00	00	1		0.543
Pit toilet	11	1 (10)	1 (0.89)	0.54	0.09–2.31	
Toilet pan	29	4 (40)	4 (3.57)	1.67	0.34–4.62	
Diaper	64	5 (50)	5 (4.46)	2.13	0.89–8.11	
Source of drinking water						
Commercial bottle water	40	1 (10)	1 (0.89)	1		0.018
Municipal water from tap	43	8 (80)	7 (7.14)	1.39	0.0823.22	
Municipal water processed at home by filter	29	1 (10)	1 (0.89)	8.91	1.0674.88	
Did you drink any local beverage made 2 weeks before? ill						
Yes	29	3 (30)	3 (2.68)	1		0.756
No	83	7 (70)	7 (6.25)	1.24	0.35–6.23	
Child nutrition within the first six months						
Exclusive breast feeding	61	2 (20)	2 (1.79)	1		0.022
No exclusive breast feeding	51	8 (80)	8 (7.14)	0.18	0.04–0.90	
Keep animals						
Yes	27	3 (30)	3 (2.68)	1		0.648
No	85	7 (70)	7 (6.25)	2.29	0.59–8.81	
Do your neighbors keep pets						
Yes	12	00		1		0.251
No	100	10 (100)	10 (8.93)	0.0	0.0- 1.0E12	

## Discussion

Out of the 112 children of ages 0–5 years with and without diarrhea recruited in this study, 10 were positive for cryptosporidiosis giving a prevalence of 8.93%. This result is less than the result obtained in Tanzania and Ethiopia [23, 24] with 10.4 and 9.4% respectively. High prevalence has also, been found in countries with high average rainfall such as Nigeria [25], 38.3%. This low prevalence is probably justified by fact that Limbe is a city with improved access to drinkable water, latrine use, less floods and good city drainage system which reduces fecal contamination rates, thus limiting occurrence of parasitosis in general and cryptosporidiosis in particular. Also, this low prevalence may be explained by methodology, using microscopy which can be less sensitive than PCR.

The prevalence amongst those that were presenting with diarrhea was 9 (13.40%).This prevalence (13.40%) is higher than the prevalence obtained in a study carried out in Rwanda (7.2%) [26], this may be due to the fact that this study was conducted mainly in the rainy season which was characterized by average rainfall, a period thus favoring the multiplication of oocysts. This prevalence (13.40%) was slightly lower than the prevalence of 16.3% obtained in Tanzania [24], where *cryptosporidium* was predominance in the rainy season and the rain cause the spread of fecal matter that contaminates drinkable water, fruit and vegetables. Also, a study carried out in Bangui, [27] among children within the ages 0–5 years was partially in line with a prevalence of Cases- 42/333 (12.6%) Controls- 9/333 (2.7%). This distribution of cryptosporidium oocyst with regards to gender, was slightly greater in females 6 (5.36%) than in males 4 (3.57%), but there was no significant difference (*p* = 0.942). The reason for these difference is not clear since they have the same exposure at crawling stage but may be probably due to the fact that the female constitute majority of the population in the study.

The prevalence of cryptosporidiosis with respect to age was not statistically significant even though prevalence 6 (5.36%) of cryptosporidiosis was obtained in the children between the age group 00–30 months old. This result is in line with a study carried out in Berlin, Germany [28] and by CDC in 2017 [29] where a higher prevalence was obtained among children of ages 00–30 months than those above 30 months. This high prevalence in this age is probably due to the fact that this age group are more vulnerable to diarrhea because basic hygiene rules are neither known nor respected and couple with the fact that the immune system is not well developed [29].

This high proportion of children with ages 0–3 years also explains why very few children in our study attended school, because the age of schooling in Cameroon is generally around 3 years. Also, this prevalence in this age group (0–5 years) can be attributed to the fact that the peak of parasitism occurs at the age at which children are sent to kindergarten and primary schools when community games and contact with dirty soil favour contamination. In addition, the rate of malnourished children in this study population 0 (00%) is lower than the rate found at the national level, which was 36% for children less than 5 years [30]. This may be due to the fact that this study was carried out in Limbe an urban area with a cosmopolitan population, where children are better fed than in rural areas where malnutrition rates may be higher. Children that never had exclusive breastfeeding within the first 6 months of life showed greater prevalence 8 (7.14%) of Cryptosporidiosis **(*p*** **= 0.022)** than in children who had exclusive breast feeding 2 (1.79%). This is probably due to the fact that most mothers, mostly the elderly in our settings consider the idea of exclusive breastfeeding as ordinary fluid from the mammary gland not being enough as a meal for an infant. Also, believing that these infants do get thirsty and require water to “quench” their thirst. Traditional force-feeding practices by forcing through oral drenching using bare hands to ensure that the children take in enough food for proper growth [31]. The risk of using bare hands (which in most cases might not be properly washed) for feeding babies may enable the transmission of food borne disease like Cryptosporidiosis from infected adult to infants. These practices generate opportunities for the ingestion of food and water contaminated with *Cryptosporidium* oocysts shed from infected individuals. This elaborates why *Cryptosporidium* is ranked 5th among the most important food borne parasites globally [31, 32].

Moreover, in most developing countries like Cameroon, *cryptosporidium* infection is common among toddler’s/younger hosts because they are more vulnerable to *Cryptosporidium* infection and it is speculated breastfeeding offers some form of protection, which may be through mother-child immunoglobulin response coupled with not using contaminated water [32]. This may explain why *Cryptosporidium* infection is mostly delayed till 6 months and beyond when complementary foods are introduced. However, the infection is common in children but tends to decrease with increasing age which suggests the development of immunity from frequent exposure to infective agents in the environment.

High prevalence 8 (7.14%) was seen in children who drank municipal water from tap**(*p*** **= 0.018)** than in children who drank commercial bottled water and municipal water processed at home by filter 1 (0.89%). This may be due to the fact that *Cryptosporidium* oocysts can resist chlorine treatment in water for months. Since the local population trust pipe borne water, it is rare to see them boil pipe borne water for the purpose of drinking. According to Medema and Schijven [25], oocysts are resistant and survive for 180 days in water and up to 1 year at 4 °C. Meanwhile, the treatment and cleaning of water treatment plants in our communities are hardly regular which could be a predisposing factor. Children 0-3 years will usually take water from unknown source and at times in dirty cups and drink. A high prevalence 8 (80%) was seen in children whose parents’ educational level was primary school. This is consistence with understanding the mode of transmission of the infection and the importance of implementing proper hygiene and sanitation practices. Majority of these parents with primary level of education turn to mostly ignore or neglect the implementation of good hygiene practices saying “black man no di die dirty”. The limitations of diagnosis of cryptosporidium based only on microscopy can underestimate or overestimate the prevalence. Since we used just microscopy due to lack of advanced methods like ELISA and PCR, our comparison of prevalence was done only with studies that used the same methodology.

## Conclusion

An overall prevalence rate of *Cryptosporidium* among children within the ages 0–5 years that attended the Limbe Regional Hospital was 8.93%. Prevalence among children that presented with diarrhea was 13.4%. Children from parents with primary level of education, children whose parents did not respect exclusive breastfeeding and those children whose parents were giving them pipe borne water for drinking recorded a higher prevalence. Also, there is a significant association between *Cryptosporidium* and diarrhea among children of ages 0–5 years presenting with diarrhea in Limbe. The results clearly demonstrated that *Cryptosporidium* is an important protozoal etiologic agent for children with diarrhea in Limbe.

## Additional file

Additional file 1: **(**QUESTIONNAIRE). (DOCX 19 kb)

## Data Availability

The datasets used and/or analysed during the current study available from the corresponding author on reasonable request.

## Abbreviations

AIDS: Acquired Immunodeficiency Virus
CDC: Cameroon Development Corporation
HIV: Human Immunodeficiency Virus
IWC: Infant Welfare Clinic
LIMIC: Low and Middle-Income Countries
mZN: Modified Ziehl Neelsen
SWR: Southwest Region

## References

[1] Checkley W, White AC, Jaganath D, Arrowood MJ, Chalmers RM, Chen XM, Fayer R, Griffiths JK, Guerrant RL, Hedstrom L, Huston CD (2015). A review of the global burden, novel diagnostics, therapeutics, and vaccine targets for cryptosporidium. Lancet Infect Dis.

[2] White ACJ, Pantenburg B, Castellanos-Gonzalez A, Dann SM, Connelly RL, Lewis DE, Ward HD (2010). Human CD8+ T cells clear Cryptosporidium parvum from infected intestinal epithelial cells. Am J Trop Med Hyg.

[3] Khan A, Shams S, Khan S, Khan MI, Khan S, Ali A (2019). Evaluation of prevalence and risk factors associated with Cryptosporidium infection in rural population of district Buner, Pakistan. PLoS One.

[4] Kotloff KL, Nataro JP, Blackwelder WC, Nasrin D, Farag TH, Panchalingam S, Wu Y, Sow SO, Sur D, Breiman RF (2013). FaruqueAS. Burden and aetiology of diarrhoeal disease in infants and youngchildren in developing countries (the global enteric MulticenterStudy, GEMS): a prospective, case-control study. LancetThe Lancet.

[5] Efstratiou A, Ongerth JE, Karanis P (2017). Waterborne transmission of protozoan parasites: review of worldwide outbreaks-an update 2011–2016. Water Res.

[6] Squire SA, Ryan U. Cryptosporidium and Giardia in Africa: current and future challenges. Parasit Vectors. 2017;10(195) https://doi.org/10.1186/s13071-017-2111-y PMID: 28427454. 10.1186/s13071-017-2111-y PMC5397716 28427454

[7] Mor SM, Tzipori S (2008). Cryptosporidiosis in children in sub-Saharan Africa: a lingering challenge. Clin Infect Dis.

[8] Shirley DA, Moonah SN, Kotloff KL (2012). Burden of disease from cryptosporidiosis. Curr Opin Infect Dis.

[9] Walker CL, Rudan I, Liu L, Nair H, Theodoratou E, Bhutta ZA, O'Brien KL, Campbell H, Black RE (2013). Global burden of childhood pneumonia and diarrhoea. LancetThe Lancet.

[10] Nahrevanian H, Azarinoosh SA, Esfandiari B, Amirkhani A, Ziapoor SP, Shadifar M (2011). The frequency of cryptosporidiosis among gastroenteritis patient’s in western cities of Mazandaran Province (2007-2009). Qazvin Univ Med Sci.

[11] Agholi M, Hatam GR, Motazedian MH (2013). HIV/AIDS-associated opportunistic protozoal diarrhea. AIDS Res Hum Retrovir.

[12] Striepen B (2013). Parasitic infections: time to tackle cryptosporidiosis. Nature News.

[13] Liu L, Li N, Xiao L, Wang L, Zhao S, Zhao X, Duan L, Guo M, Feng Y (2012). Molecular surveillance of Cryptosporidium spp., Giardiaduodenalis, and Enterocytozoon bieneusi by genotyping and subtypingparasites in wastewater. PLoS Negl Trop Dis.

[14] Aghamolaie S, Rostami A, Havildar Biderouni F, Haghighi A (2016). Evaluation of modified Ziehl–Neelsen, direct fluorescent-antibody and PCR assay for detection of Cryptosporidium spp. in children faecal specimens. J Parasit Dis.

[15] Tellevik MG, Moyo SJ, Blomberg B, Hjøllo T, Maselle SY, Langeland N, Hanevik K (2015). Prevalence of Cryptosporidium parvum/hominis, Entamoeba histolytica and Giardia lamblia among young children with and without diarrhea in Dar Es Salaam, Tanzania. PLoS Negl Trop Dis.

[16] Nsagha DS, Njunda AL, Assob NJ, Ayima CW, Tanue EA, Kwenti TE (2015). Intestinal parasitic infections in relation to CD4+ T cell counts and diarrhea in HIV/AIDS patients with or without antiretroviral therapy in Cameroon. BMC Infect Dis.

[17] Bissong ME, Nguemain NF, Ng'awono TE, Kamga FH (2015). Burden of intestinal parasites amongst HIV/AIDS patients attending Bamenda regional Hospital in Cameroon. Afr J Clin Exp Microbiol.

[18] Vouking MZ, Enoka P, Tamo CV, Tadenfok CN. Prevalence of intestinal parasites among HIV patients at the Yaounde Central Hospital, Cameroon. Pan Afr Med J. 2014;18. 10.11604/pamj.2014.18.136.3052 PMC4236773 25419274

[19] Sarfati C, Bourgeois A, Menotti J, Liegeois F, Moyou-Somo R, Delaporte E, Derouin F, Ngole EM, Molina JM (2006). Prevalence of intestinal parasites including microsporidia in human immunodeficiency virus–infected adults in Cameroon: a cross sectional study. Am J Trop Med Hyg.

[20] Shah UV, Purohit BC, Chandralekha D, Mapara MH (2005). Co-infection with cryptosporidium, isospora and S. stercoralis in a patient with AIDS- a case report. Indian J Med Microbiol.

[21] Nassar SA, Oyekale TO, Oluremi AS (2017). Prevalence of Cryptosporidium infection and related risk factors in children in Awo and Iragberi, Nigeria. J Immunoass ImmunochemJournal of Immunoassay and Immunochemistry.

[22] Anejo-Okopi JA, Okojokwu JO, Ebonyi AO, Ejeliogu EU, Isa SE,Audu O, Akpakpan EE, Nwachukwu EE, Ifokwe CK, Ali M and LarP. Molecular characterization of cryptosporidium in children aged 0-5years with diarrhea in Jos, Nigeria. Pan Afr Med J. 2016;12(3):25. 10.11604/pamj.2016.25.253.10018 PMC5337289 28293369

[23] Nasser AM (2016). Removal of Cryptosporidium by wastewater treatment processes: a review. J Water Health.

[24] Kabayiza JC, Andersson ME, Nilsson S, Bergström T, Muhirwa G, Lindh M (2014). Real-time PCR identification of agents causing diarrhea in Rwandan children less than 5 years of age. Pediatr Infect Dis J.

[25] Medema GJ, Schijven JF (2001). Modelling the sewage discharge and dispersion of Cryptosporidium and Giardia in surface water. Water Res.

[26] United Nations Children's Fund, World Health Organization, World Bank Group. Levels and Trends in Child Malnutrition—UNICEF/WHO/World Bank Group Joint Child Malnutrition Estimates, 2017.

[27] Breurec Sébastien, Vanel Noémie, Bata Petulla, Chartier Loïc, Farra Alain, Favennec Loïc, Franck Thierry, Giles-Vernick Tamara, Gody Jean-Chrysostome, Luong Nguyen Liem Binh, Onambélé Manuella, Rafaï Clotaire, Razakandrainibe Romy, Tondeur Laura, Tricou Vianney, Sansonetti Philippe, Vray Muriel (2016). Etiology and Epidemiology of Diarrhea in Hospitalized Children from Low Income Country: A Matched Case-Control Study in Central African Republic. PLOS Neglected Tropical Diseases.

[28] Chalmers RM, Cacciò S. Towards a consensus on genotyping schemes for surveillance and outbreak investigations of Cryptosporidium, Berlin, June 2016. Euro surveillance. 2016;21(37). 10.2807/1560-7917.ES.2016.21.37.30338 PMC5032853 27685759

[29] www.cdc.gov/parasite; Last updated 8, 2017, Accessed 20,7,2018.

[30] Nasser AM. Removal of Cryptosporidium by wastewater treatment processes: a review. J WaterHealth. 2016;14(1):1-3. 10.2166/wh.2015.131 26837825

[31] Aniesona AT, Bamaiyi PH (2014). Retrospective study of cryptosporidiosis among Diarrhoeic children in the arid region of north-eastern Nigeria. Zoonoses Public Health.

[32] Ryan UN, Fayer R, Xiao L (2014). Cryptosporidium species in humans and animals: current understanding and research needs. Parasitology..

