# Supplementary material for: Prevalence and risk factors associated with cryptosporidiosis among children within the ages 0–5 years attending the Limbe regional hospital, southwest region, Cameroon
Source: BMC Public Health. 2019 Aug 20;19:1144. doi: 10.1186/s12889-019-7484-8 (PMC6700837; doi:10.1186/s12889-019-7484-8)
Supplement: Supplementary file 1 — (QUESTIONNAIRE). (DOCX 19 kb) [file 12889_2019_7484_MOESM1_ESM.docx]

**Additional files**

**Additional file 1 (**QUESTIONNAIRE)

QUESTIONNAIRE

Dear Participants,

I am ATSIMBOM NEVILLE TOMBANG, a second year M.Sc. student of Medical Microbiology and Parasitology, Department of Medical Laboratory Science from the Faculty of Health Science in the University of Buea. I will be carrying out a research study on “Prevalence and Risk factors associated with Cryptosporidiosis among children 0-5 years attending Limbe Regional Hospital, S.W.R. Cameroon”. This work is in partial fulfilment for the award of M.Sc. in Medical Microbiology and Parasitology. All the information given here will be treated with utmost confidentiality.

SECTION A: PATIENT’S INFORMATION.

1) Gender: (1) MALE (2) FEMALE

2) Code/Full

name……………………………………………………………………………..

3) Age:………………………………………………………………………………

4) Residence:…………………………………..Duration in the locality:………………

5) Parent/caregiver’s Phone Number………………………………………………………

SECTION B: ECONOMIC STATUS OF PARENTS/CAREGIVER

6) Level of education. (1) Primary (2) Secondary (3) University

7) Occupation: (1) Farming (2)

Others(specify)……………………………………

8) Monthly income estimate : (1) <20.000F (2) 20-50.000F (3) 50-100.000F (4) 100-150.000F (5) >150.000F

9) What type of residence are you living in now? (1) Private home (2) Rented (3) Camp houses

10)House type: (1) Cement Block house (2) Plank house

11)How many individuals currently live in your household?.................................

SECTION C: CLINICAL INFORMATION

12) How long have you had these symptoms?....................................................

13) Have you had any of the following symptoms? (1) Diarrhea (2) Nausea

(3) Vomiting (4) Fever (5) Loss of weight or appetite (6)Cramping (7) Gas (8) Headache (9) Other (specify)………………………………………………………

14) On any regularly medication before your symptoms began? (1) YES (2) NO

15) If Yes, Tell me the name of this (these) medications…………………………………

16) Do your child has a weakened immune symptom? ( In other words, is the child HIV positive, receiving cancer chemotherapy, or an organ transplant recipient?) (1) YES (2) NO (3) Unknown

SECTION D: EXPOSURE INFORMATION

17) Infant/child Nutrition within the first 6months: (1) Exclusive breastfeeding (2) Breastfeeding & Complementary feed practices 18) Is the child schooling? (1) YES (2) NO

19) Is the child using toilet by him/herself? (1) YES (2) NO

20) Type of toilet; (1) Flushing toilet (2) pit’s toilet (3) Others…………………..

21) What are your sources of drinking water at home? (1) Municipal water from the tap (2) Municipal water processed with a home filter (3) Well water (4) Commercially bottled water (5) Others (specify)………………………………………

22) What are your sources of drinking water at school? (1) Municipal water from the tap (2) Municipal tap water filtered at home and taken to school (3) Well water (4) Commercially bottled water (5) Others (specify)……………

23) During the 2 weeks before you became ill, did you drink any local beverage made with water, such as folere, yogout, etc ? (1) YES (2) NO

24) If Yes, name of local
beverage……………………………………………………….

25) During the 2 weeks before you became ill, did you swim in a pool, lake or river? (1) YES (2) NO

26) If Yes, Where did you swim?................................................................

27) Do you remember accidentally swallowing any of the water? (1) YES (2) NO

28) During an average week, how many meals do you order/buy and bring home, including breakfast, lunch, and dinner? NUMBER OF MEALS………….

29) Did this child in the last 2 weeks drank unpasteurized milk, unpasteurized apple juice, and/or eat any unpasteurized products? (1) YES (2) NO

30) What unpasteurized product(s) did you eat?.............................................

31) Do this child attend a child care center? (1) YES (2) NO

32) Do you rear animals where you live ? (1) YES (2) NO

33) If yes, (1) Goat (2) Sheep (3) Cattle (4) Dogs (5) Fowls (6) Others (specify)…

34) Do you visit a vetinary Doctor regularly? (1) YES (2) NO

35) If Yes do you properly dispose their stool ? (1) YES (2) NO

36) Do your close neighbors have pets around the compound you live ? (1) YES (2)NO
